# Molecular Patho-mechanisms of cervical cancer (MMP1)

**DOI:** 10.1016/j.amsu.2022.103415

**Published:** 2022-03-31

**Authors:** Iwan Kurnia, Syahrul Rauf, Mochammad Hatta, Sharvianty Arifuddin, Yudi Maulana Hidayat, Rosdiana Natzir, Cahyo Kaelan, Agussalim Bukhari, Nugraha Utama Pelupessy, Ilham Jaya Patelonggi

**Affiliations:** a Department of Obstetrics and Gynaecology, Faculty of Medicine, Hasanuddin University, Makassar, Indonesia; b Department of Molecular Biology and Immunology, Faculty of Medicine, Hasanuddin University, Makassar, Indonesia; c Department of Obstetrics and Gynaecology, Faculty of Medicine Universitas Padjajaran, Bandung, Indonesia; d Department of Biochemistry, Faculty of Medicine, Hasanuddin University, Makassar, Indonesia; e Department of Anatomical Pathology, Faculty of Medicine, Hasanuddin University, Makassar, Indonesia; f Department of Clinical Nutrition, Faculty of Medicine, Hasanuddin University, Makassar, Indonesia; g Department of Physiology, Faculty of Medicine, Hasanuddin University, Makassar, Indonesia

## Abstract

Cervical cancer mostly caused by Human Papilloma Virus. Staging and therapy have been extensively studied, and highly correlated with the cellular development of oncogenesis. Mutation was caused by E6 and E7 oncoprotein, also inactivation of 2 tumor suppressor factors (pRB and p53). P53 also regulated MMP1, which dysregulation of MMP transcription would promote tumor metastasis, because of its role in extracellular matrix degradation in tumor invasion. Clinical staging of Cervical Cancer was based on *Federation International of Gynaecology and Obstetrics* (FIGO) classification from 2018. Management was divided into Surgery, Radiotherapy, and Chemotherapy.

## Highlights

• Metalloproteinase from a matrix, known as Matrixin or MMP (Matrix Metallo Proteinase), was part of sub-group of zinc-endoproteinase, produced by soft tissue.
• This enzyme then involved in an important cascade that resulted in soft tissue degradation, either physiologically or pathologically.
• Matrix metalloproteinases (MMPs) have two opposite functions, which are promoting and inhibition of cancer.
• Physiologically, MMP expression was very low or even zero in all of human's tissues. It only increased in reactive or reparative condition.

## 1 Introduction

Cervical cancer is a type of neoplasm in the cervix, mostly caused by Human Papilloma Virus (HPV). Anatomically, cervix is one third lower portion of uterus, cylindrical, protruding, and connected with vagina through external orifice of the uterus. Risk of cancer could be caused by genetic factors, bad lifestyles habit, less hygiene, and sexually active with multiple partners [1] (see Table 1, Table 2, Pic 1, Pic 2, Pic 3, Pic 4)

**Table 1:** FIGO Staging of cervical cancer.

Stage	Description
I	The carcinoma is strictly confined to the cervix (extension to the uterine corpus should be disregarded)
IA	Invasive carcinoma that can be diagnosed only by microscopy, with maximum depth of invasion <5 mm
IA1	Measured stromal invasion depth of <3 mm
IA2	Measured stromal invasion depth 23 mm and <5 mm
IB	Invasive carcinoma with measured deepest invasion of 25 mm (greater than Stage IA), lesion limited to the cervix uteri
IB1	Invasive carcinoma with measured deepest stromal invasion of 25 mm, and greatest dimension of <2 cm
IB2	Invasive carcinoma with greatest dimension of 22 cm and <4 cm
IB3	Invasive carcinoma with greatest dimension of >4 cm
IB	Invasive carcinoma with measured deepest invasion of 25 mm (greater than Stage IA), lesion limited to the cervix uteri
IB1	Invasive carcinoma with measured deepest stromal invasion of 25 mm, and greatest dimension of <2 cm
IB2	Invasive carcinoma with greatest dimension of 22 cm and <4 cm
IB3	Invasive carcinoma with greatest dimension of >4 cm
II	The carcinoma invades beyond the uterus, but has not extended into the lower third of the vagina or to the pelvic wall
IIA	A Involvement limited to the upper two-thirds of the vagina without parametrial invasion
IIA1	Invasive carcinoma with greatest dimension of <4 cm
IIA2	Invasive carcinoma with greatest dimension of ≥4 cm
IIB	With parametrial involvement but not up to the pelvic wall
III	The carcinoma invades beyond the uterus, but has not extended into the lower third of the vagina or to the pelvic wall
IIIA	T3 III T3a A The carcinoma involves the lower third of the vagina, with no extension to the pelvic wall
IIIB	Extension to the pelvic wall and/or hydronephrosis or nonfunctioning kidney (unless known to be due to another cause)
IIIC	Involvement of pelvic and/or para-aortic lymph nodes, irrespective of tumor size and extent (with r and p notations)
IIIC1	Pelvic lymph node metastasis only
IIIC2	Para-aortic lymph nodes metastasis
IV	The carcinoma has extended beyond the true pelvis or has involved (biopsy proven) the mucosa of the bladder or rectum (the presence of bullous edema is not sufficient to classify a case as Stage IV)
IVA	Spread to adjacent pelvic organs
IVB	Spread to distant organs

**Table 2:** Histopathological classification of cervical cancer.

1. Squamous carcinoma • Keratinizing • Large cell non keratinizing • Small cell non keratinizing • Verrucous
2. Adeno carcinoma • Endocervical • Endometroid (adenoacanthoma) • Clear cell - paramesonephric • Clear cell - mesonephric • Serous • Intestinal
3. Mixed carcinoma • Adeno-squamous • Mucoepidermoid • Glossy cell • Adenoid cystic
4. Undifferentiated carcinoma
5. Carcinoma tumor
6. Malignant melanoma
7. Malignant non-epithelial tumors • Sarcoma: mixed Mullerian, leiomyosarcoma, rhabdomyosarcoma • Lymphoma

75% of cervical cancer were squamous cell carcinoma.

10–15% were adenocarcinoma, the rest were other types.

**Pic 1:**
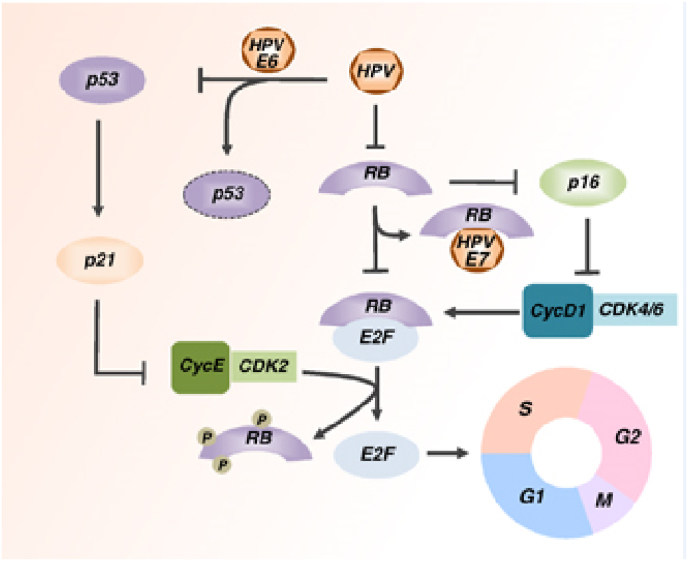
HPV Mechanism of Action in deactivating Tumor Suppressor Gene [2].

**Pic 2:**
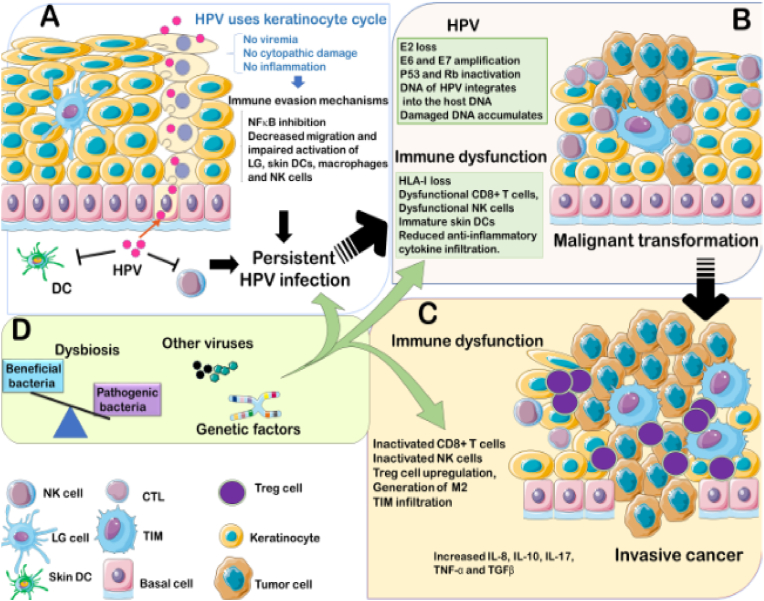
Carcinogenesis in persistent HPV [3]→16.

**Pic 3:**
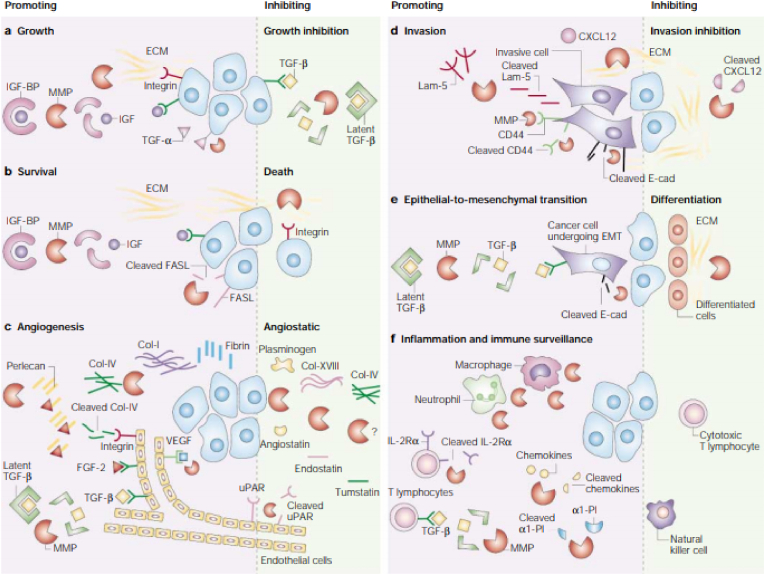
MMP1 role in Cancer Progressivity [4].

**Pic 4:**
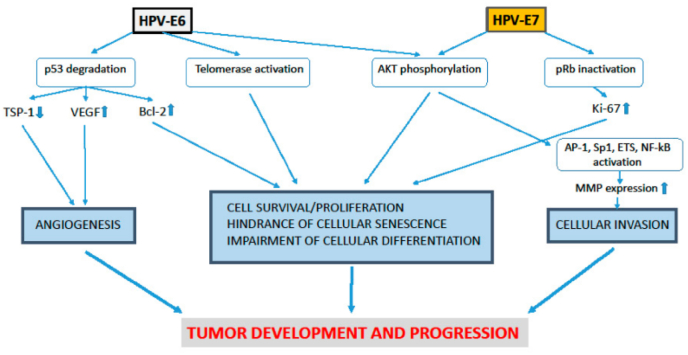
MMP1 role in tumor development and progressiveness [5].

More than 2 million of ≥15 years old women are at risk of cervical cancer. 527624 are diagnosed each year with 265672 death. Cervical cancer is 4th most common cancer in women, and second highest in women between age 15–44. In Asia alone, the incidence was 284823. Whereas Southeast region had 9082 for age 15 to 39, 32892 cases for age 40–64, and 8581 cases for women above 65 year of age. Indonesia ranked 4th highest case in Southeast Asia, after Cambodia, Myanmar, and Thailand. Around 7.9% (528000) new cases dan 7.5% (266000) death are reported in 2012. More than 85% of cervical cancer cases and 87% of deaths are reported in the less developed countries. It was the second most cause of death after breast cancer, mostly due to lower early-detection rate of precancerous lesion [6].

Based on Health Ministry Data in 2005, cancer incidence was 1 in 1000 each year, 15000 new cervical cancer cases every year with 7500 cancer-related death. According to Cancer Registration by Indonesian Pathology Association in 2011, Cervical Cancer had ranked second highest cancer in women in Indonesia with 3023 cases and more than 75% were presented as advanced stage. In RSCM, year 2015, there were 443 cases with 68.2% were presented as advanced stage [7].

### 1.1 Cervical Cancer

Cervical cancer is a very progressive disease, started with intraepithelial lesion, neoplastic, then cancerous after 10 years or more. In Histopathology, pre-invasive lesion usually was developing through different stages of dysplasia (mild, moderate, severe) into *karsinoma in situ*, then invasive lesion. In general principal of carcinogenesis, cancerous process started with mutation of gene that controls cell-cycle, which are oncogenes, tumor suppressor genes, and repair genes. Oncogenes mediate malignant transformation and tumor suppressor genes works in the opposite ways. It's true that cancer started with intraepithelial lesion, but it didn't always progress into invasive lesion [8].

Most of the cervical cancer were caused by chronic infection of human papilloma virus (HPV) high-risk oncogene sub-types [9]. HPV is a double-stranded circular DNA virus with 8000 base and 55 nm in diameter. The carcinogenicity of HPV is mediated by oncogenes activity of E (early gene) 6 and E7. E6 is corelated with tumor suppressor p53, and E7 with pRb. There're more than 120 HPV sub-types out of 30 types that infected squamous epithelia of lower anogenital tract. 15 of those types are classified as definitive carcinogenic, which are types 16, 18, 31, 33, 34, 35, 39, 45, 51, 52, 56, 58, 59, 66, and 68. HPV was detected in 99.7% of cervical cancer cases [10,11].

HPV DNA has 8 open reading frames (ORFs), which are the early region that consist of E1, E2, E4, E5, E6, E7 (expressed at early differentiation), L1 and L2 (expressed at the end of differentiation) and control area (long control region) that located between E and L. All ORF sequence code produce one genome strand, that's divided functionally into 3 regions. First, noncoding upstream regulatory region, also called noncoding region, long control region (LCR), or upper regulation region. This consist of 11 promotors of p97, enhancer and silencer that controlled DNA replication. Second, Early Region (45%), consist of ORF (open reading frames) E1, E2, E4, E5, E6, and E7, in viral replication and oncogenesis. Third, the late region (40%) consists of L1 (95%) and L2 (5%) which were structural protein in HPV capsid [12,13].

E2 holds an important role in the regulation of viral replication, it binds directly to the DNA chromosome. This bond disrupts E2 expression, which in turn increase E6 and E7 expression. E1 and E2 have roles in the coding of protein that control E6 and E7 genes (part of oncoprotein) in viral replication. E1 and E2 also play a role in viral transcription. E4 is a coding protein strand that plays a role in viral growth and maturation. E5 induce loss of expression of MHC (Major Histocompatibility Complex)-I in epitheliums, enabling HPV to hinder host immunity in the early stage of differentiation (code for hydrophobic protein that produce immortal cell). E5 as a weak oncogene also play a role in increasing EGFR (epidermal growth factor receptor) which suppress MHC expression [14]. E6 and E7 of HPV are the main oncogenic protein, especially in cancerous process because of the ability to bind and degrade tumor suppressor gene p53 and pRb in the infected host cells. These tumor suppressor genes work to break cell-cycle and cell proliferation. E6 binds to cell associated protein (AP) and E6-AP complex would damage and causing Tumor suppressor gene (TSG) p53 to loss its function. This damage decrease cell-cycle check point and apoptosis, then cell proliferation grow out of control. Whereas E7 binds to TSG pRb, causing loss of E2F. Without E2F as a transcription factor, cell cycle will also get uncontrolled growth. Each protein has a target, especially retinoblastoma tumour suppression protein (pRB). E6 inhibits apoptosis from p53, whereas E7 inhibits resting cell-cycle [15]. E6 also induce secretion of Vascular Endothelial Growth Factor (VEGF). L1/L2 play role in coding of structural protein in virus functioning and completion of viral particle formation. Besides, HPV DNA has LCR, that regulate E6 and E7 transcription. Thus, both E6 dan E7 were closely related to carcinogenesis in cervical cancer [15,16]. 12 High risk HPV types were causing genetic instability through metilation of cellular DNA [17], (proto)oncogene activity, tumour suppressor genes (TSG) deactivation, dan telomerase activity [18].

85% of HPV infection could resolve spontaneously without therapy, but 15% persist by immune escape, weakened antiviral activity of keratinocyte; antigen presenting cell (APCs); immune response of macrophage and natural killer (NK) cells; reducing apoptosis, etc. [19].

Main risk factors were younger age and multiple partners sexual activity. First sexual activity at under 20 years of age increase cervical cancer risk by 8-fold, and multiple partners increase it by 4-fold [20].([21]).

Nutritional factors, education and economy level could also increase the risk. Malnutrition correlate with decrease of immune to defense against HPV. Micronutrient such as carotenoid, folate, vitamin C, lycopene and cryptoxanthin have protective effect in NIS1 to regress back to normal growth. Patient with low immune caused by chronic infection such as n HIV (Human Immunodeficiency Virus) or other chronic infection could increase progression of precancerous to cancerous lesion [22].

Multi parity increase the risk of cervical squamous cell carcinoma in women with positive HPV infection [23,24].Nubia Munoz et all reported a direct corelation between number of births with cervical squamous cell carcinoma, in which the OR (Odd Ratio) was 7-fold than nullipara women by 3.8 (95% CI: 2.7–5.5) and 2.3 (95% IK 1,6-3,2) in women with 1–2 births. Risk of adenocarcinoma or adeno-squamous was not correlated with the number of births [25].

Smoking has a high correlation with HPV infection. Tobacco consists of carcinogens, either inhaled which produce polycyclic aromatic hydrocarbons heterocyclic amine; or chewed that produce nitrosamine. This correlation was reflected by RR (Relative Risk) of 1.50 with 95% CI: 1.35–1.66 but did not increase the risk of adenocarcinoma (RR: 0.86; 95% CI: 0.70–1.05) [26].(54(21) [27].

Other sexually transmitted infection such as *Chlamydia trachomatis* (CT) and Herpes simplex virus type 2 (HSV-2) also increase cervical cancer risk in women with HPV positive. These may be caused by inflammation of cervix that induce genotoxic damage through reactive oxidative metabolite. Case control analysis in multiple studies showed positive correlation with OR 1.8 (95% CI: 1.2–2.7) [28].(22).

Oral contraception also increases cervical cancer risk. Compared to women without history of oral contraception, the risk in patients using oral contraception was increasing in accordance with duration of usage. RR for duration under 5 years, 5–9 years, and 10 years or more were 1.1 (95% CI: 1.1–1.2), 1.6 (95% CI: 1.4–1.7), and 2.2 (95% CI: 1.9–2.4), respectively for all women; dan 0.9 (95% CI: 0.7–1.2), 1.3 (95% CI: 1.0–1.9), and 2.5 (95% CI: 1.6–3.9) for women with HPV infection. One of the meta-analysis reported that cervical cancer risk increased in the group with more than 5 years of usage, compared with groups with no history of usage (RR 1.9; 95% CI: 1.69–2.13). The risk decreased after stop using; and after 10 years or more, the risk would be the same as the group without history of usage [[29], [30], [31]].([32]).

Gold standard of Cervical Cancer diagnosis is based on histopathological examination of cervical biopsy. Early stage of cancer usually symptomatic, but some of the common symptoms were([33]):

a. Vaginal bleeding
b. Increasing and bad odor leukorrhea
c. Pain: usually in intermediate and advanced stage, or infected neoplasm. Located at lower abdomen, gluteal region, or sacrococcygeal. Lower abdominal pain could indicate infection, water accumulation, or pus in the uterine cavity, causing uterus contraction and pain. Intermittent pain could be caused by tumor compression or invasion that obstruct or dilate ureter. Hydronephrosis might cause low back pain, lower extremities, gluteal, or sacrum pain; also due to tumor pressure to nerve of pelvic cavity region.
d. Urinary tract symptoms (often due to infection): incontinence, urgency, dysuria. With cancer progression to bladder, hematuria and pyuria developed, even cysto-vaginal fistula. When cardinal ligament or ureter was invaded, hydronephrosis and uremia ensued.
e. Digestive problems: lesion could spread to cardinal or sacral ligament, put pressure on rectum, causing obstipation; even invaded rectum and lead to hematochezia and rectovaginal fistula.
f. Systemic symptoms: weakness, lethargy, fever, weight loss, anemia, and edema.

Staging is important in determining disease spread, prognosis, management plan, and comparing therapeutic methods. Clinical staging was based on *Federation International Of Gynaecology And Obstetrics* (FIGO) classification from 2018 [34].

Squamous carcinoma were the most common types, which were accounted for ± 90%, adenocarcinoma 5%, and others 5% [35].

After diagnosis was confirmed by histopathology and the stage was set by clinical manifestation and radiology, management was started based on the location, tumor size, stage, patient's age, general conditions, and fertility reservation.

1. Surgery

Surgery could stand as curative and palliative care. Curative is therapy to eliminate causes and clinical manifestarion of the diseases. Whereas palliative is a way to correct patient's condition with or without eliminating the cause. Radical hysterectomy has goals to remove uterus and cervix, parametrium, paracolpium, and vagina. It was usually done at the early stage, which was IA until IIA (FIGO Classification).

2. Radiotherapy

Radiation could destroy cancer cells in the cervix, parametrium, pelvic wall, and lymph nodes. It was recommended for stage IIB, III, and IV. Like surgery, it also has a curative and palliative purposes. In curative, radiation destroy metastatic cancer cells in lymph nodes while preserves as much as possible healthy tissues in rectum, urinary bladder, small bowel, and ureter. It was done in the stage IIB until IIIC, or even at the earlier stage when there was contraindication to surgery or as an adjuvant therapy after surgery with high re-occurrence rate. When cancer has spread outside pelvic wall, palliative radiation was given selectively in stage IVA. Radiation used high energy ray to destroy and hamper cancer growth. Side effect would be rectal and vaginal irritation, damage to urinary bladder, rectum, and ovarium.

3. Chemotherapy

Route of administration of chemotherapy as a management of cancer, could be oral, intravenous, intraperitoneal, or intramuscular. The primary goals were to kill cancer cells, hamper growth, or as a radiosensitiser. Based on cancer type and stage, it could act as curative (especially in facility without radiotherapy) or palliative.

Study by Rameri (2017) with neoadjuvant chemotherapy in cervical cancer stage IB2-IVA showed that the overall recurrency rate (ORR) was not that different than the cases with definitive treatment [36,37].

Purposes of neoadjuvant chemotherapy were to decrease tumor size to facilitate surgery, decrease recurrence rate and increase survival rate. But it was controversial, based on the study. In patient that resistant to chemotherapy, it would delay the definitive therapy. Study in Prague also showed that complete response rate to neoadjuvant chemotherapy was 12.6% with progressive rate of 6%, thus it was very important to have a certain marker that could show which patient might be resistant to chemotherapy [38].

Clinical factors such as age, haemoglobin level (Hb), tumor size, histopathology cancer type, and differentiation degree, would affect chemotherapy response, as well as angiogenesis factors as a response to hypoxia in cervical cancer [[39], [40], [41]].

Hypoxia was reported to play a role in resistance to chemotherapy and radiotherapy. Hypoxic cell released HIF (hypoxia inducible factor), which in turn induce angiogenesis factors, such Vascular Endothelial Growth Factor (VEGF), angiopoietin, angiogenin, and Platelet Derived Growth Factor (PDGF). Then they activated endothelial protease, proliferation, and migration, as well as reducing apoptosis activity [42].

As cancer spread in the advanced phase, chemotherapy act more in palliative care, to preserve patient's quality of life. Combination chemotherapy was used in metastatic diseases as single agent would not give satisfied effect. Most common combination were platinum and taxane.

Cervical Cancer has a bad prognosis, because 85–90% were diagnosed at invasive, advanced, or even terminal stage. Parameter in determining prognostic factors were clinical and histopathological, such as: general condition, staging, primary tumor size, cell types, and Broders differentiation degree. Generally, 5-years survival rate for Stage I was more than 90%, 60–80% for Stage II, around 50% for stage III, to less than 30% for stage IV.

1 Stage 0: 100% of patients will recover.
2 Stage 1: divided into IA and IB. IA has 5-years survival rate of 95%. As for stage IB, 5-years survival rate were 70–90%. Women with cancer in lymph node were not included.
3 Stage 2: divided into 2A and 2B. 2A has 5-years survival rate of 70–90%. And 5-years survival rate of stage 2B were 60–65%.
4 Stage 3: 5-years survival rate were 30–50%.
5 Stage 4: 5-years survival rate were 20–30%.

### 1.2 MMP-1

Metalloproteinase from a matrix, known as Matrixin or MMP (Matrix Metallo Proteinase), was part of sub-group of zinc-endoproteinase, produced by soft tissue. This enzyme then involved in an important cascade that resulted in soft tissue degradation, either physiologically or pathologically. Decrease of extracellular matrix (ECM) was part of an important step in invasion and metastasis of neoplasm. This resulted from activation of matrix metalloproteinase (MMPs) that degraded protein component of ECM, followed by other physiological process, such as angiogenesis, apoptosis, and new soft tissue production. All these would support the development of cancer [43].

MMP also known to be correlated with formation and development of squamous cell carcinoma (SCC). MMP gene involved in SSC, includes MMP1, 2, 3, 7, 9, 10, 11, 12, 13, which over-expressed in the SCC tissues, compared to normal tissue. Each of those genes could be classified into different categories. MMP1 was part of collagenase in interstitial [44].

As interstitial collagenase, abnormal expression of MMP1 was seen in the development of cancer. Over-expression was clearly detected in several cancer cases, and highly correlated with prognosis. Besides, MMP1 also promoted angiogenesis by activating protease-activated receptor1 in endothelial [45].

MMP1 that's produced by tumor cells contributed functionally in hematogenous spread of SCC. It induced vascular permeability through activation of endothelial Protease Activated Receptor (PAR)-1, thus made invasion and metastasis possible [45].

Matrix metalloproteinases (MMPs) have two opposite functions, which are promoting and inhibition of cancer.

A. MMPs promote cancer growth by *cleaving insulin-growth-factor-binding protein* (IGF-BP), releasing IGF; through transmembrane precursor *growth factors* including *growth factor-α* (TGF-α); and by regulating extracellular matrix, indirectly increase interaction between extracellular matrix and integrins. At the other hand, MMP could also slowed down cancer growth through Transforming *growth factor-β* (TGF-β) from latent TGF-β complexes.
B. MMP increase cancer survival by IGF initiation through FAS ligand pathway (FASL), that play a role in *death receptor* FAS. But MMPs could also cause apoptosis, by changing extracellular matrix composition, that influence integrin signal.
C. MMPs promote angiogenesis by increasing bioavailability of pro-angiogenesis *vascular endothelial growth factor* (VEGF), *fibroblast growth factor 2* (FGF-2), and TGF-β. These stimulated endothelial proliferation and migration. In addition, MMPs also induce cancer invasion through extracellular matrix structural component, such as: Collagen type I (Col-I) and IV (Col-IV) as well as fibrin. Collagen played role as pro-angiogenesis by binding with integrin αvβ3. At the other hand, MMPs also have a role as anti-angiogenesis through plasminogen and Col-XVIII, producing angiostatin and endostatin factors. MMPs participate in *urokinase-type plasminogen activator receptor* (uPAR) on cell surface.
D. MMPs regulate invasion by degrading extracellular matrix structural component, especially through laminin 5 (Lam-5) pathway, CD44 molecule adhesion pathway, and E cadherin (E-cad). In addition, cancer cell invasion needs MMP-9 to CD44 migration. But MMPs could inhibit metastasis through CXCL12 pathway. On the contrary, chemokine of CXC family could promote breast cancer metastasis.
E. MMPs promote *epithelial-to-mesenchymal transition* (EMT), correlated with malignancy through molecular pathway E-cad dan TGF-β. MMPs increased differentiation by changing extracellular matrix component and integrin signal.
F. Cellular inflammation was the main key in MMPs involvement in cancer progress because it also inhibits immune reaction towards cancer cells. MMPs broke down interleukin-2-α (IL-2Rα) receptor on T-lymphocytes, which in turn inhibit T-lymphocytes proliferation; releasing TGF-β, which is an important suppressor of T-cell reaction against cancer cell; cleave *α1-proteinase inhibitor* (α1-PI), decreasing cancer sensitivity toward *natural killer cells*; and split CC and CXC chemokine family, that help cancer cells slip away from leukocytes.

**Figure:**
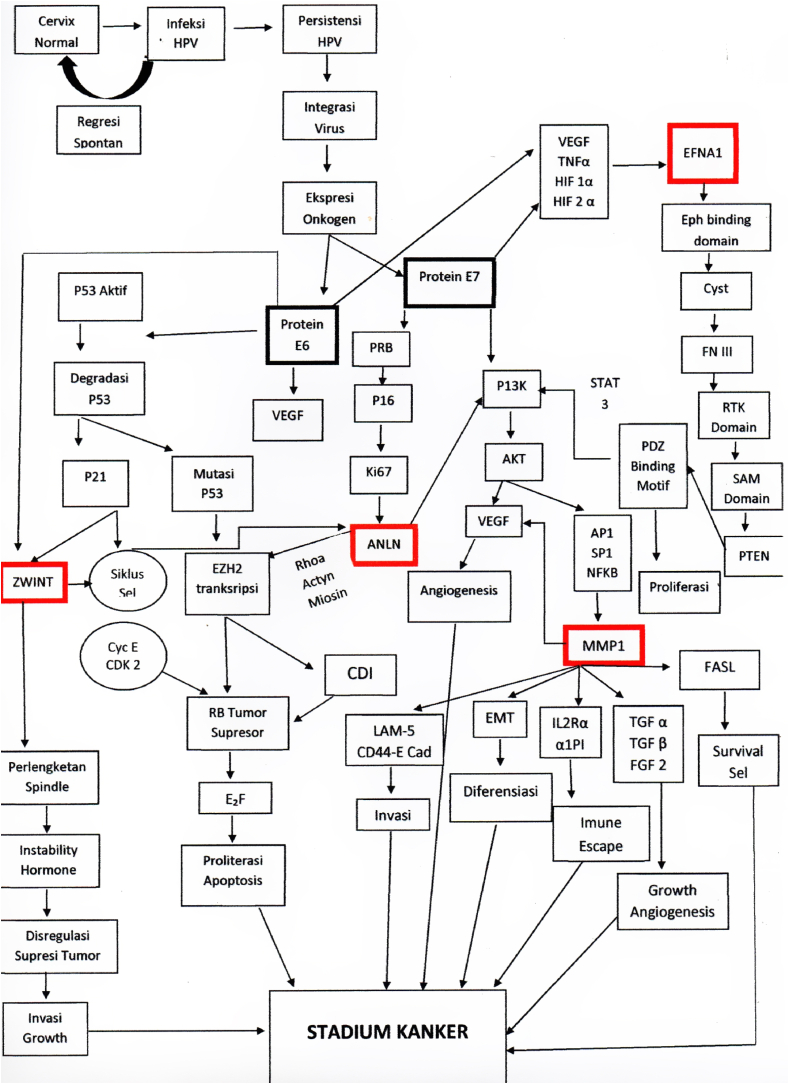

Multiple factors contributed to the development of oncogenesis. Changes in signalling pathways, accompanied by genetic instability and mutation werw caused by high-risk Human Papilloma Virus (HPV) infection (E6 and E7 oncoprotein) [46]. Besides those high-risk oncoproteins, most important mechanism was inactivation of 2 tumor suppressor factors (pRB and p53) [47].

E6 was able to degrade p53 by direct contact with E6AP ubiquitin ligase, thus inhibiting p53-dependant signal and contributing to tumorigenesis. E7 could interact with retinoblastoma protein groups (pRb, p107, and p130) and disrupt E2F groups of transcription factors. These unplanned interactions caused *trans*-activation of cellular protein needed for viral DNA replication. Persistent high-risk HPV infection induce HPV integration into the host genome, causing overexpression of E6 and E7. Interaction with DNMT in turn, caused deviation of tumor suppressor gene metilation. This is the main factors in carcinogenesis [47].

E6 effect in multiple pathways of carcinogenesis would influence initiation, progression, and metastasis. E6 activated PIK3 through AKT pathway. It also had effect on PTEN by activation PDZ protein, which in turn increase pAKT and cell proliferation. These indicated increase in ribosomal protein S6 kinase [48] Akt phosphorylases E6 and promoted its ability to interact with 14-3-3σ that has an important role in carcinogenesis [49]. E6 could also increase telomerase reverse transcriptase (TERT) regulation that coded human telomerase reverse transcriptase (hTERT). These processes stabilized NFX1-123 interaction and increased telomerase expression [50].

E7 interaction with HDAC resulted in chromosome remodelling and genome instability. E7 also activated PIK3 or AKT pathway. It correlated with its ability to bind and activate Rb protein, and in turn correlate with HPV-induced high-grade Intraepithelial squamous lesion [51].

Cell cycles depend on cyclin protein and Cyclin-dependent kinase (CDKs). Cyclin regulate CDKs. CDKs became active when it binds with cyclin and formed complexes. Cyclin was classified into A, B, D and E, each played a role in different point of cell cycles. Cyclin D were synthesized at the beginning of G1 phase, bound with CDK4 and CDK6. At the end of G1, Cyclin E were synthesized and bound with CDK2. When 3 complexes were formed, cell enter S phase. This holds important role in the initiation of DNA replication (Jackson etc, 1995) and cell cycle transition [[3], [20]]. Cyclin E strongly held chromatin, thus capable of hindering replication. In mitosis, this complex was blocked by phosphorylated Cyclin E, that was recycled at the end of mitosis, enabling new cycle of DNA replication [52].

In transcription regulation, Cyclin E has a receptor site for transcription factor E2F. This induced transcriptional repression by binding with large complexes containing E2F4, DP1, and protein socket that repressed Cyclin E expression till the end of G1 phase. pRb could also formed large repressor complexes with Histone Deacetylase (HDAC) and mammalian complex component hSW1/snf, like SNF-2(BRG1 and hbrm), start at the end of S phase to G1 phase [53]).

hSW - SNF complex interacted with cyclin E and modulated BRG1 ability to restrain cell growth [54] This held important role in transcription regulation, chromatin structural change by erasing transcription using nucleosome-mediated repression. Thus, opening access of transcription activator [26]

pRb phosphorylation by cyclin D/CDK4 cancelling its interaction with HDAC and cyclin E transactivation, ending G1 phase Cyclin A and CDK1 transcription were withheld by pRb and hSW/SNF complexes. Cyclin E/CDK2 could phosphorylate pRb or hSW/SNF components when cyclin E concentration were high enough and pRb and hSW/SNF interaction was disrupted. Disregulation of each transcription complexes happened in cancer cell. Studies showed that there's overexpression of Cyclin E when Rb was inactivated in HPV16 infection with E7 oncogene [28] Although most induction factor was E2F. Cyclin E also increased in p53 mutation, by endogen as well as transfection [55].

Cancer was mentioned as a disease caused by dysregulation of cell proliferation [56] Cell programmed death or apoptosis was very important in preventing tumor growth, thus dysregulation of this mechanism would promote neoplasm (143)(144). Defect of this pathway could be Rb gene deletion or dysregulation of CDH that phosphorylate and inactivate Rb [57].

P53 also regulated MMP1 (147)→57, which were a zinc-bonded endopeptidase in human. Dysregulation of MMP transcription would promote tumor metastasis [58], because of its role in extracellular matrix degradation in tumor invation 59) [[59], [60], [61]], Studies showed that MMP1 was one of the proteins that's overexpressed in various cancer [62]) [[63], [64], [65]].

Physiologically, MMP expression was very low or even zero in all of human's tissues. It only increased in reactive or reparative condition [[66], [67], [68], [69]] MMP function were actively regulated by globulin and endogenous tissue inhibitors of MMP (TMMP) [[66], [67], [68]] Cancer cells could synthesize MMP after oncogene activation, inactivation of onco-suppressor, stimulated by growth factor or inflammation mediator, reactive oxygen species or hypoxia [69,70].

MMP holds an important role in tumor growth development. Specifically, MMP digested molecule on cell surface that mediated cell adhesion with other cells or ECM. Thus promoting cancer cell penetration in hematogenous and lymphatic spread, then metastasis [71].

Eph receptors were consist of 3 parts [72], which were:

1. Extracellular domain, incl. connective tissue domain, rich in cysteine and fibronectin type III
2. Transmembrane domain
3. Intracellular domain

Unlike Eph, Ephrin-A didn't have intracellular domain that attached to cell-membrane through glycosyl lipo-inositol groups [72]. Eph receptor and Ephrin bond produced two-way signal that's attached to the cell. These regulated cell structure, migration, defence, and proliferation [[73], [74], [75]]Forwarding signal could activate STAT3 and PIK 3/1 KT pathway in various tumor, thus promoting cancer cell migration and invasion [[76], [77], [78], [79], [80]]. Those could also cause endocytosis and proteolysis. Signal transduction of Ephrin-A was caused by interaction of glycosylphosphatidylinositol groups with transmembrane domain [74,[81], [82], [83]],

Abnormal expression of Eph and Ephrin in tumor cells correlated with cancer growth, metastasis, and tumor spread, as well as host survival (180)(181). [84,85], The expression also regulated by transcription factor in oncogenic signal, metilation promoter and microRNA [75,86]. EphA12 was one of the most common dysregulated expression in tumor cells. Such as melanoma, glioma, breast cancer, prostatic, lungs, and cervical cancer [[87], [88], [89], [90]] EphA2 overexpression was highly correlated with other tumor-related signalling pathway activation, for example AKT/mTOR, RAS/MAPK, and WNT/beta catein [[91], [92], [93]],
